# Epidemic of Mumps among Vaccinated Persons, the Netherlands, 2009–2012

**DOI:** 10.3201/eid2004.131681

**Published:** 2014-04

**Authors:** Jussi Sane, Sigrid Gouma, Marion Koopmans, Hester de Melker, Corien Swaan, Rob van Binnendijk, Susan Hahné

**Affiliations:** National Institute for Public Health and the Environment, Bilthoven, the Netherlands (J. Sane, S. Gouma, M. Koopmans, H. de Melker, C. Swaan, R. van Binnendijk, S. Hahné);; European Programme for Intervention Epidemiology Training, European Centre for Disease Prevention and Control, Stockholm, Sweden (J. Sane);; Erasmus Medical Centre, Rotterdam, the Netherlands (S. Gouma, M. Koopmans)

**Keywords:** mumps, outbreaks, epidemic, immunization, surveillance, MMR, vaccine, vaccination, the Netherlands, viruses, herd immunity

## Abstract

To analyze the epidemiology of a nationwide mumps epidemic in the Netherlands, we reviewed 1,557 notified mumps cases in persons who had disease onset during September 1, 2009–August 31, 2012. Seasonality peaked in spring and autumn. Most case-patients were males (59%), 18–25 years of age (67.9%), and vaccinated twice with measles-mumps-rubella vaccine (67.7%). Nearly half (46.6%) of cases occurred in university students or in persons with student contacts. Receipt of 2 doses of vaccine reduced the risk for orchitis, the most frequently reported complication (vaccine effectiveness [VE] 74%, 95% CI 57%–85%); complications overall (VE 76%, 95% CI 61%–86%); and hospitalization (VE 82%, 95% CI 53%–93%). Over time, the age distribution of case-patients changed, and proportionally more cases were reported from nonuniversity cities (p<0.001). Changes in age and geographic distribution over time may reflect increased immunity among students resulting from intense exposure to circulating mumps virus.

Mumps is an acute illness caused by mumps virus (family *Paramyxoviridae*) and characterized by fever, swelling, and tenderness of >1 salivary gland, usually the parotid gland. Complications associated with mumps include orchitis (inflammation of the testes), meningitis, pancreatitis, and deafness. Mumps virus is spread in respiratory droplets, and the incubation period is 15–24 days (median 19) (*1*). 

Vaccination for mumps has been in use in industrialized countries for decades (*2*). The Netherlands began mumps vaccination in 1987, using the measles, mumps, and rubella combination vaccine (MMR). The vaccine, containing the Jeryl-Lynn mumps virus strain, is administered in a 2-dose schedule at 14 months and 9 years of age. Vaccination coverage of >1 dose of MMR has consistently been >93% since the introduction of the vaccination program (*3*). After the MMR program was launched, the incidence of mumps in the Netherlands decreased considerably; nevertheless, during the 2000s, several mumps outbreaks were detected. In 2004, an outbreak occurred among students at an international school (*4*), and in 2007–2008, an outbreak was detected mainly in a religious community that had low vaccination coverage (*5*). Since the end of 2009, a countrywide epidemic has been ongoing, affecting mainly student populations (*6*,*7*).

Mumps was notifiable in the Netherlands before 1999 and was made notifiable again in December 2008 (*5*). Mumps surveillance reports are released biweekly or monthly and include data on age and sex distribution, geographic distribution, vaccination, and contact status of case-patients. The report is distributed to public health professionals, including epidemiologists, virologists, and local-level health professionals, but comprehensive spatiotemporal characterization of the surveillance data has not been conducted. To provide information for future mumps prevention efforts, we used this surveillance data to assess the rates of illness and complications associated with the ongoing outbreak, to understand who is at risk for infection, and to assess whether transmission patterns have changed over time.

## Methods

We reviewed data on mumps cases reported to the registration system for notifiable infectious diseases in the Netherlands (OSIRIS) during September 1, 2009–August 31, 2012. Notification criteria for mumps include >1 related symptom (i.e., acute onset of painful swelling of the parotid or other salivary glands, orchitis, or meningitis) and laboratory confirmation of infection or an epidemiologic link to a laboratory-confirmed case (*7*). In addition to basic demographic information, notification data reported to OSIRIS included vaccination status and student or contact with student status. The questions on student/student contact status were made more specific on April 19, 2010. For cases reported before that date, the information for the new variable was obtained from open-format questions. Laboratory confirmation criteria included >1 of the following: detection of mumps-specific IgM; detection of viral RNA; or isolation of the virus on cell culture. Genotyping targeting the gene encoding the small hydrophobic protein was performed on specimens submitted to the National Institute for Public Health and the Environment by using an in-house method. 

We used the χ^2^ test for comparison of proportions and testing for trends over time and calculated a 3-week moving average to characterize trends and seasonality. Vaccine effectiveness (VE) was estimated as 1 – odds ratio. The odds ratio, which describes the association between complications/hospitalizations and vaccination status, was adjusted for age and sex (when outcome was orchitis, adjustment was done for age only) and estimated by using logistic regression. Associations with p values of <0.05 were considered statistically significant, and all reported p values are 2-tailed. Stata software version 12 (StataCorp, College Station, TX, USA) was used for the analyses.

## Results

During September 1, 2009–August 31, 2012, a total of 1,557 cases of mumps were reported in the Netherlands (Figure); 1,254 (80.5%) of these were laboratory confirmed. Laboratory confirmation was most often by detection of viral RNA (68.8%), followed by antibody detection (21.9%) and virus isolation (7.3%). In 2% of cases, 2 methods were combined for diagnosis. 

**Figure:**
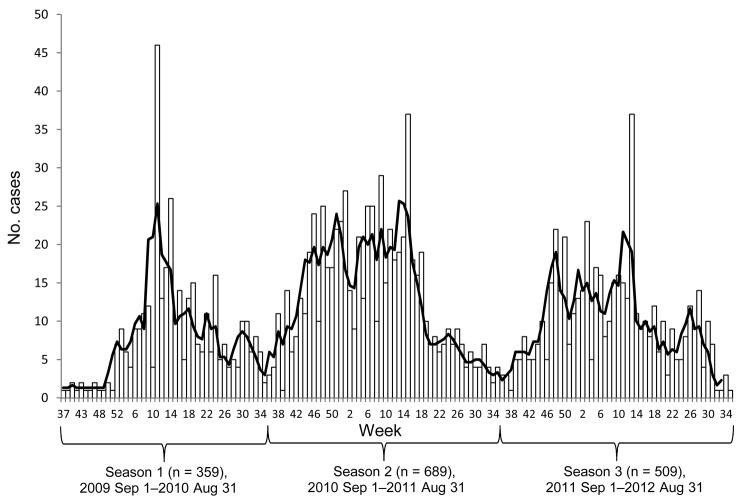
Numbers of notified mumps cases, by week of onset, The Netherlands, September 1, 2009–August 31, 2012 (N = 1,557 cases). Seasons and number of cases (n) are indicated; black line indicates 3-week moving average.

Most case-patients were male (59%) and 18–25 years of age (67.9%). The average annual incidence per 100,000 population was 0.5 for the 0–3-year age group, 0.8 for the 4–14-year age group, 4.5 for the 13–17-year age group, 21.4 for the 18–25-year age group, and 0.9 for the >25-year age group. Of the 1,474 cases for which patient vaccination status was reported, 998 (67.7%) case-patients had received 2 doses of MMR; 157 (10.6%) had received 1 dose, and 242 (16.4%) were unvaccinated. Genetic analysis of small hydrophobic gene sequences of 808 mumps-positive samples showed that most (98.5%) outbreak strains belonged to the G5 subtype.

Complications were reported in 126 cases (8.4% of 1,492 cases with known complication status) (Table 1). Most (78 [62%]) complications occurred in the 18–25-year age group. Orchitis was the most frequent complication (109 [12.7%] male case-patients >12 years of age) and occurred significantly more often among unvaccinated case-patients than among case-patients who had received 1 vaccine dose (p = 0.04); vaccination with 2 doses of MMR reduced the risk for orchitis even further (p<0.01). Other reported complications were meningitis (n = 6), pancreatitis (n = 3), thyroiditis (n = 1), and encephalitis (n = 1). Three case-patients had permanent unilateral hearing loss that was probably caused by mumps virus infection. Deafness and meningitis occurred more frequently among unvaccinated than vaccinated persons, but those numbers were probably too low for statistical significance (Table 1). 

**Table 1:** Association between rates of mumps complications and hospitalization and MMR status, the Netherlands, September 1, 2009–August 31, 2012*

**Complication**	**No. MMR doses received by case-patient**	**No. (%) case-patients with complications**	**Crude OR (95% CI)**	**p value**	**aOR† (95%CI)**	**p value**	**aVE† (95% CI)**
Orchitis§	0	36 (15.5)	Ref		Ref	Ref	Ref
	1	10 (6.6)	0.46 (0.22–0.97)	0.04	0.46 (0.22–0.98)	0.04	54 (2–78)
	2	46 (4.7)	0.26 (0.16–0.41)	<0.01	0.26 (0.15–0.43)	<0.01	74 (57–85)
Deafness	0	2 (0.9)	Ref				
	1	0	NA	NA	–	–	–
	2	1 (0.1)	0.12 (0.01–1.3)	0.1	–	–	–
Meningitis	0	2 (0.8)	Ref				
	1	1 (0.6)	0.76 (0.07–8.5)	0.8	–	–	–
	2	2 (0.2)	0.24 (0.03–1.7)	0.2	–	–	–
All complications	0	44 (19.0)	Ref		Ref	Ref	Ref
	1	10 (6.6)	0.30 (0.15–0.62)	<0.01	0.29 (0.14–0.62)	<0.01	71 (38–86)
	2	55 (5.7)	0.26 (0.17–0.39)	<0.01	0.24 (0.14–0.39)	<0.01	76 (61–86)
Hospitalization	0	11 (4.8)	Ref		Ref	Ref	Ref
	1	3 (2.0)	0.41 (0.11–1.5)	0.18	0.43 (0.11–1.6)	0.2	57 (–60 to 89)
	2	10 (1.1)	0.22 (0.09–0.52)	<0.01	0.18 (0.07–0.47)	<0.01	82 (53–93)

*Only case-patients with known complications and vaccination status were included in the analyses. OR, odds ratio; VE, vaccine effectiveness; Ref, referent; NA, not applicable; –, not analyzed (insufficient sample size). †Adjusted for age (age groups <18 y, 18–25 y, >25 y) and sex, except orchitis, which was adjusted only for age. §Includes only male case-patients >12 y of age.

A total of 31 patients (2.1% of 1,436 patients with known hospitalization status) were hospitalized. Risk for hospitalization was significantly lower among case-patients who had received 2 MMR doses than for unvaccinated case-patients (p<0.01); VE for preventing hospitalization was 82% (Table 1). Of the 31 hospitalized case-patients, 13 (42%) had orchitis. No deaths were reported.

Three distinct epidemic seasons occurred during the outbreak: seasonal peaks in spring and late autumn and a decline in number of cases during summer and, to some extent, during the Christmas holidays (Figure). Data on sex, age, vaccination status, residence in a city with a university, student status, and contact with student status by season are shown in Table 2. Overall, the age distribution of mumps case-patients differed significantly between the seasonal peaks (p = 0.007). The number of cases increased proportionally over time for the 13–17-year age group (p = 0.003) and the >25-year age group (p = 0.042) and decreased over time for the 18–25-year age group (p<0.001). The overall proportion of cases in vaccinated persons did not change (Table 2), and the proportion of complications or hospitalizations did not differ by season (data not shown).

**Table 2:** Demographic characteristics and student status for 1,557 patients with mumps, by annual epidemic season, the Netherlands, September 1, 2009–August 31, 2012*

**Characteristic**	**No. (%) case-patients**		
	Season 1, 2009 Sep 1–2010 Aug 31, n = 359	Season 2, 2010 Sep 1–2011 Aug 31, n = 689	Season 3, 2011 Sep 1–2012 Aug 31, n = 509
Sex			
M	205 (57.1)	416 (60.4)	296 (58.2)
F	154 (42.9)	271 (39.3)	213 (41.8)
Unknown	0	2 (0.3)	0
Age, y			
0–3	3 (0.8)	4 (0.6)	3 (0.6)
4–12	5 (1.4)	22 (3.2)	16 (3.1)
13–17	17 (4.7)	63 (9.1)	**54 (10.6)**
18–25	270 (75.2)	468 (67.9)	**318 (62.4)**
>25	64 (17.8)	131 (19)	**118 (23.2)**
Unknown	0	1 (0.2)	0
Vaccination status			
0 doses	57 (15.9)	115 (16.7)	70 (13.7)
1 dose	37 (10.3)	69 (10.0)	51 (10.0)
2 doses	225 (62.7)	436 (63.3)	337 (66.2)
>3 doses	4 (1.1)	4 (0.6)	5 (1.0)
Vaccinated but unknown no. doses	24 (6.7)	25 (3.6)	15 (3.0)
Unknown	12 (3.3)	40 (5.8)	31 (6.1)
Residence in a city with university†			
Yes	258 (71.9)	351 (50.9)	**243 (47.7)**
No	92 (25.6)	322 (46.7)	**263 (51.7)**
Unknown	9 (2.5)	16 (2.3)	3 (0.6)
Student/contact with students			
Not a student and no contact with students	22 (6.1)	171 (24.8)	**118 (23.2)**
University student or contact with university students	229 (63.8)	275 (39.9)	**221 (43.4)**
Other student‡ or contact with other students	20 (5.6)	144 (20.9)	**88 (17.3)**
Unknown	88 (24.5)	99 (14.4)	82 (16.1)
Incidence estimates§			
University students	92.9	93.9	80.2
Other students	2.0	14.7	9.8
Secondary school students	0	0.7	5.4

*Boldface indicates significance trends by χ^2^ test, calculated by using proportions excluding unknowns. †University cities: Amsterdam, Delft, Eindhoven, Enschede, Groningen, Leiden, Maastricht, Nijmegen, Rotterdam, Stichtse Vecht, Tilburg, Utrecht, and Wageningen. ‡Students enrolled in higher education other than university. §Incidence per 100,000 students. Total student numbers by category obtained from www.cbs.nl.

We found significant seasonal differences in the proportion of cases occurring in students and in persons with student contacts (p<0.001). During early spring 2010, large clusters of cases were reported from university cities of Leiden and Delft, as described (*6*). However, during 2011 and 2012, proportionally more case-patients were not students and had no contact with students than during 2010 (p<0.001). The proportion of student case-patients enrolled in higher education other than university or case-patients who had contact with these nonuniversity students increased after 2010 (p<0.001). The absolute numbers of cases in these categories increased from 2010 to 2011 but stayed more or less constant, or decreased slightly, in 2012. The number of case patients who were university students or who had contact with university students decreased proportionally (p<0.001), and over time, proportionally more cases were reported from cities without universities (p<0.001). In addition, the total number of cases from nonuniversity cities was higher in 2012.

## Discussion

The epidemic of mumps in the Netherlands during late 2009 through 2012 affected mainly vaccinated students. However, vaccination evidently offered protection against mumps-associated complications. The epidemic showed a seasonal trend, although cases were identified throughout the years. Over time, age, student status, and geographic distribution changed, which suggests a slight shift in transmission trends from student populations to younger and older nonstudent populations and to cities without a university. This shift may relate to increased immunity in the primarily affected high-risk student population; exposure to wild-type mumps virus may have boosted individual immunity and thus contributed to increased herd immunity.

Mumps outbreaks among vaccinated populations have been reported in other countries during recent years: a 2006 outbreak in the United States (*8*), a 2009–2010 outbreak in Canada (*9*), and a 2012 outbreak in the United Kingdom (*10*). Description of an outbreak in 2009–2010 in the northeastern United States among a highly vaccinated population of Orthodox Jews indicated that intense exposure among boys in a religious school facilitated the transmission of mumps virus, which overpowered the vaccine-induced protection (*11*,*12*). Similar to our findings, transmission in that outbreak shifted from adolescents to younger and older populations over time. The intense social crowding among students (e.g., large indoor social gatherings) partly explains why secondary vaccine failure occurred in the outbreak described in this study. A subgroup of students, including those living with many other students and members of university fraternities, may be at increased risk for infection (*6*,*7*). Crowding in nonstudent populations may not be as intense as among students, and mixing is usually with more heterogeneous age groups. In these circumstances, herd immunity is sufficient to prevent more widespread transmission. A lower rate of crowding may be one explanation for the relatively low numbers of cases among 4–12-year-olds, despite the generally lower IgG titers in this group than in adolescent students. (*13*). Still, even though lower antibody levels do not automatically mean higher risk for mumps virus infection (*14*), a higher rate of illness would have been expected in the 4–12-year age group. An additional explanation for the lower apparent illness rate among these younger children might be a higher frequency of unapparent and subclinical infections, which would lead to many undiagnosed cases in this age group.

Most of the persons affected in the epidemic were male, a finding also observed in other studies (*15*,*16*). The reasons for male predominance are unclear, but significantly higher mumps antibody titers in female than in male persons have been demonstrated (*13*,*17*); this finding, in turn, may be linked to gender-associated genetic differences in immune response. Behavioral differences between sexes may also play a role.

Most cases occurred in persons who had received 2 doses of MMR, which suggests inadequate effectiveness of the vaccine. Recent studies indicate the effectiveness of MMR against mumps is moderate and lower than the clinical efficacy estimates (*1*,*18*). Postlicensure studies of 2 doses (Jeryl-Lynn strain) of MMR have provided a median VE estimate of 88% (range 79%–95%) (*2*). A recent study of an outbreak of mumps at a student party in the Netherlands estimated a VE of 68% for 2 doses of MMR (*6*). This estimate is, however, uncertain because of the low number of unvaccinated case-patients. We attempted to provide VE estimates against clinical mumps applying the screening method; however, because this method is most vulnerable to error when proportions of the population and case-patients vaccinated are high (*19*), as in this study, the estimates became inaccurate and thus are not included in our results. The possible causes for lower than expected VE include secondary vaccine failure (waning immunity), intense exposure to high virus inoculum, and a possible mismatch between the vaccine genotype and circulating strains (*1*,*2*,*18*,*20*). However, because the level of antibodies correlating with protection remains unknown (*12*,*21*), we are unable to further elucidate the role of these factors.

Orchitis was the most common complication, consistent with previous outbreaks in a population with a similar age structure (*1*). However, orchitis occurred significantly more often among unvaccinated than vaccinated case-patients, and the vaccine was effective in preventing orchitis, which has previously been shown in a study based in part on the same study population (*22*) and in other studies (*11*,*23*). Vaccination also significantly reduced the risk for complications overall and for hospitalizations. A previous report described 3 cases of deafness (0.19% of all notified infections), 2 in unvaccinated persons (*24*). The frequency of 0.005% for unilateral deafness commonly cited in the literature (*25*) is considerably lower than that found in our study, but this difference is likely attributable to a different denominator population. A higher incidence of deafness has been reported from Japan using more appropriate denominators (*26*). 

One limitation of our study was the short time span for assessing changes over time. Mumps cases have continued to occur after our study period, but the number of cases reported after September 2012 (180 as of August 31, 2013) is much lower than that reported during the previous years. Recent numbers indicate that a similar trend in changing patterns of age and geographic distribution is ongoing; most of the more recent cases have occurred in nonstudents and in age groups other than 18–25 years (data not shown). However, because of lower case numbers, this comparison must be interpreted with caution. 

A further limitation of our study is that it is likely that many mumps cases are not notified because they are subclinical infections or because of reluctance to seek medical care; thus, these cases are not included in our analyses. Furthermore, complications that occurred after the notification date are not included; however, because vaccination status is probably not associated with the reporting of complications, we regard our VE estimates against complications as unbiased.

Although VE for mumps vaccination is not optimal for preventing clinical disease, our results support previous findings that vaccination limits the severity of disease. Because complications are the primary mumps-associated public health problem, these findings support the current vaccination recommendations. Still, this epidemic demonstrates that mumps virus can cause large outbreaks even in highly vaccinated populations. The observation that the incidence after the third season studied has been considerably lower than during previous seasons is consistent with the development of herd immunity among high-risk students resulting from the high rate of natural symptomatic and asymptomatic infections. However, the annual inflow of new susceptible students—unvaccinated and vaccinated—who start their studies could again lower overall immunity. A recent study suggested that use of a third MMR dose might be an effective control measure in certain outbreak situations (*27*). Introduction of a third MMR dose to the vaccination schedule has been considered in the Netherlands (*6*) but was not recommended because of relatively low overall illness rates associated with mumps and other factors, including an expected low vaccine uptake. Although the vaccine remains effective in most settings and significantly reduces the risk for complications, further research is needed to understand the limitations of MMR, and modeling is warranted to understand the dynamics of mumps virus transmission in future.

## References

[1] Hviid A, Rubin S, Muhlemann K. Mumps. Lancet. 2008;371:932–44. 10.1016/S0140-6736(08)60419-5 18342688

[2] McLean HQ, Hickman CJ, Seward JF; World Health Organization Department of Immunization, Vaccines and Biologicals. The immunological basis for immunization series. Module 16: mumps. Geneva: The Organization; 2010 [cited 2014 Jan 10]. http://whqlibdoc.who.int/publications/2010/9789241500661_eng.pdf

[3] van Lier EA, Oomen PJ, Oostenbrug MW, Zwakhals SL, Drijfhout IH, de Hoogh PA, High vaccination coverage of the National Immunization Programme in the Netherlands [in Dutch]. Ned Tijdschr Geneeskd. 2009;153:950–7 . 19490720

[4] Brockhoff HJ, Mollema L, Sonder GJ, Postema CA, van Binnendijk RS, Kohl RH, Mumps outbreak in a highly vaccinated student population, the Netherlands, 2004. Vaccine. 2010;28:2932–6. 10.1016/j.vaccine.2010.02.020 20188683

[5] Snijders BE, van Lier A, van de Kassteele J, Fanoy EB, Ruijs WL, Hulshof F, Mumps vaccine effectiveness in primary schools and households, the Netherlands, 2008. Vaccine. 2012;30:2999–3002. 10.1016/j.vaccine.2012.02.035 22381073

[6] Greenland K, Whelan J, Fanoy E, Borgert M, Hulshof K, Yap KB, Mumps outbreak among vaccinated university students associated with a large party, the Netherlands, 2010. Vaccine. 2012;30:4676–80. 10.1016/j.vaccine.2012.04.083 22579874

[7] Whelan J, van Binnendijk R, Greenland K, Fanoy E, Khargi M, Yap K, Ongoing mumps outbreak in a student population with high vaccination coverage, Netherlands, 2010. Euro Surveill. 2010;15:19554 . 20460086 10.2807/ese.15.17.19554-en

[8] Cortese MM, Jordan HT, Curns AT, Quinlan PA, Ens KA, Denning PM, Mumps vaccine performance among university students during a mumps outbreak. Clin Infect Dis. 2008;46:1172–80. 10.1086/529141 18444852

[9] Deeks SL, Lim GH, Simpson MA, Gagne L, Gubbay J, Kristjanson E, An assessment of mumps vaccine effectiveness by dose during an outbreak in Canada. CMAJ. 2011;183:1014–20. 10.1503/cmaj.101371 21576295 PMC3114893

[10] Calvert N, Ashton JR, Garnett E. Mumps outbreak in private schools: public health lessons for the post-Wakefield era. Lancet. 2013;381:1625–6. 10.1016/S0140-6736(13)60953-8 23628443

[11] Barskey AE, Schulte C, Rosen JB, Handschur EF, Rausch-Phung E, Doll MK, Mumps outbreak in Orthodox Jewish communities in the United States. N Engl J Med. 2012;367:1704–13. 10.1056/NEJMoa1202865 23113481

[12] Plotkin SA. Correlates of protection induced by vaccination. Clin Vaccine Immunol. 2010;17:1055–65. 10.1128/CVI.00131-10 20463105 PMC2897268

[13] Smits G, Mollema L, Hahné S, de Melker H, Tcherniaeva I, Waaijenborg S, Seroprevalence of mumps in the Netherlands: dynamics over a decade with high vaccination coverage and recent outbreaks. PLoS ONE. 2013;8:e58234. 10.1371/journal.pone.0058234 23520497 PMC3592917

[14] Jokinen S, Osterlund P, Julkunen I, Davidkin I. Cellular immunity to mumps virus in young adults 21 years after measles-mumps-rubella vaccination. J Infect Dis. 2007;196:861–7. 10.1086/521029 17703416

[15] Hassan J, Dean J, Moss E, Carr MJ, Hall WW, Connell J. Seroepidemiology of the recent mumps virus outbreaks in Ireland. J Clin Virol. 2012;53:320–4. 10.1016/j.jcv.2011.12.022 22269391

[16] Cohen C, White JM, Savage EJ, Glynn JR, Choi Y, Andrews N, Vaccine effectiveness estimates, 2004–2005 mumps outbreak, England. Emerg Infect Dis. 2007;13:12–7 . 17370510 10.3201/eid1301.060649 PMC2913658

[17] Ovsyannikova IG, Jacobson RM, Dhiman N, Vierkant RA, Pankratz VS, Poland GA. Human leukocyte antigen and cytokine receptor gene polymorphisms associated with heterogeneous immune responses to mumps viral vaccine. Pediatrics. 2008;121:e1091–9. 10.1542/peds.2007-1575 18450852 PMC2668976

[18] Dayan GH, Rubin S. Mumps outbreaks in vaccinated populations: are available mumps vaccines effective enough to prevent outbreaks? Clin Infect Dis. 2008;47:1458–67. 10.1086/591196 18959494

[19] Farrington CP. Estimation of vaccine effectiveness using the screening method. Int J Epidemiol. 1993;22:742–6. 10.1093/ije/22.4.742 8225751

[20] Quinlisk MP. Mumps control today. J Infect Dis. 2010;202:655–6. 10.1086/655395 20662719

[21] Cortese MM, Barskey AE, Tegtmeier GE, Zhang C, Ngo L, Kyaw MH, Mumps antibody levels among students before a mumps outbreak: in search of a correlate of immunity. J Infect Dis. 2011;204:1413–22. 10.1093/infdis/jir526 21933874

[22] Hahné S, Whelan J, van Binnendijk R, Swaan C, Fanoy E, Boot H, Mumps vaccine effectiveness against orchitis. Emerg Infect Dis. 2012;18:191–3. 10.3201/eid1801.111178 22260843 PMC3381682

[23] Yung CF, Andrews N, Bukasa A, Brown KE, Ramsay M. Mumps complications and effects of mumps vaccination, England and Wales, 2002–2006. Emerg Infect Dis. 2011;17:661–7. 10.3201/eid1704.101461 21470456 PMC3377415

[24] Opstelten W, Hahné SJ, van Roijen JH, van Paridon L, Wolters B, Swaan CM. Mumps makes a comeback [in Dutch]. Ned Tijdschr Geneeskd. 2012;156:A5187 . 23095482

[25] Everberg G. Deafness following mumps. Acta Otolaryngol. 1957;48:397–403. 10.3109/00016485709126900 13508195

[26] Hashimoto H, Fujioka M, Kinumaki H. An office-based prospective study of deafness in mumps. Pediatr Infect Dis J. 2009;28:173–5. 10.1097/INF.0b013e31818a8ca8 19209100

[27] Ogbuanu IU, Kutty PK, Hudson JM, Blog D, Abedi GR, Goodell S, Impact of a third dose of measles-mumps-rubella vaccine on a mumps outbreak. Pediatrics. 2012;130:e1567–74. 10.1542/peds.2012-0177 23129075

